# Supplementary material for: Artificial Gravity Attenuates the Transcriptomic Response to Spaceflight in the Optic Nerve and Retina
Source: Int J Mol Sci. 2024 Nov 9;25(22):12041. doi: 10.3390/ijms252212041 (PMC11593819; doi:10.3390/ijms252212041)
Supplement: Supplementary file 1 [file ijms-25-12041-s001.zip › Supplementary File S1.pdf]

ON 0G

R1

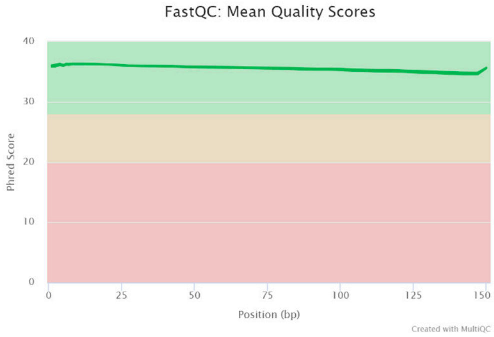

R2

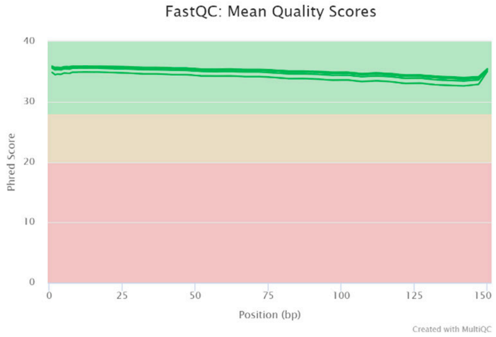

ON 0.33G

R1

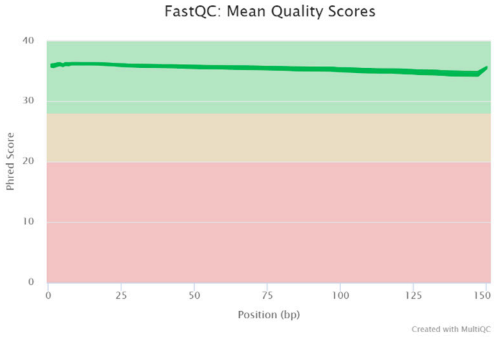

R2

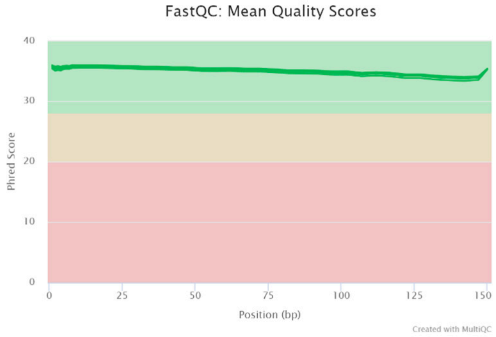

ON 0.67G

R1

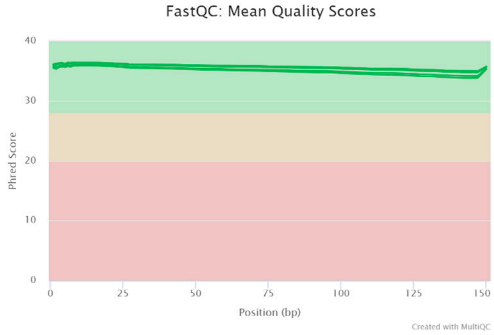

R2

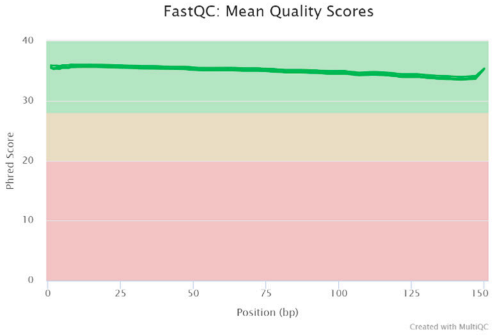

ON 1G

R1

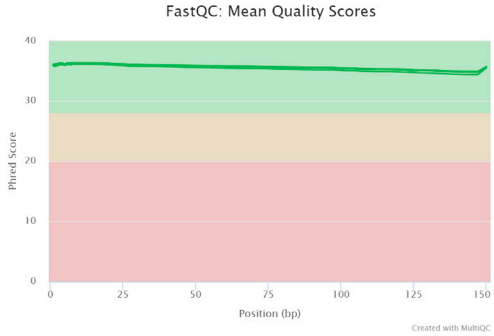

R2

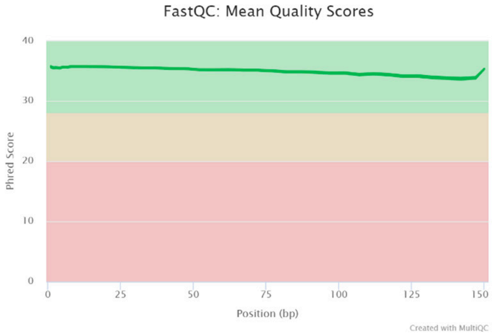

ON HGC

R1

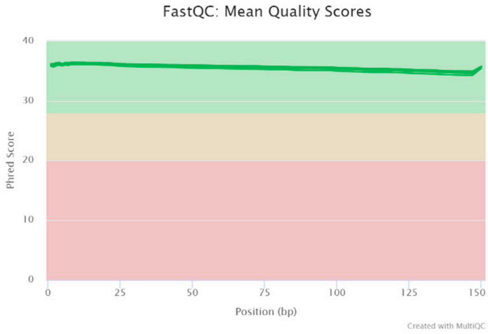

R2

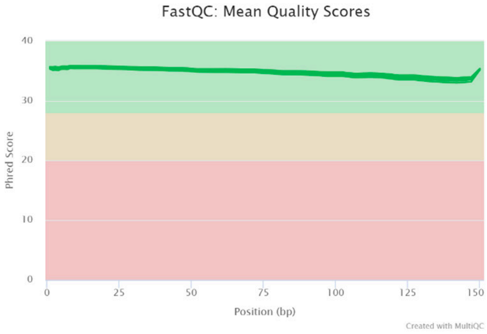

(a) MultiQC Analysis ON

RTN 0G

R1

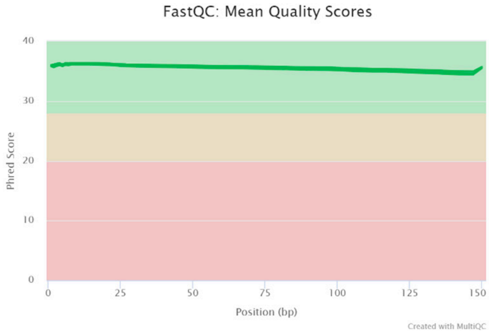

R2

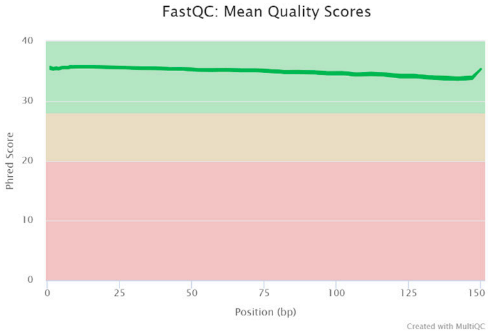

RTN 0.33G

R1

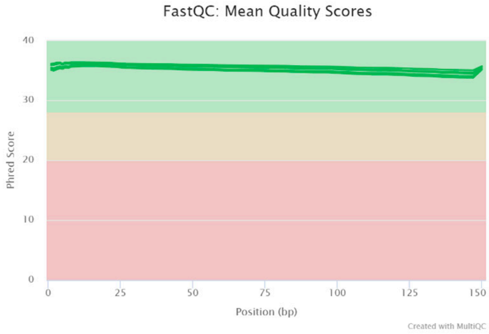

R2

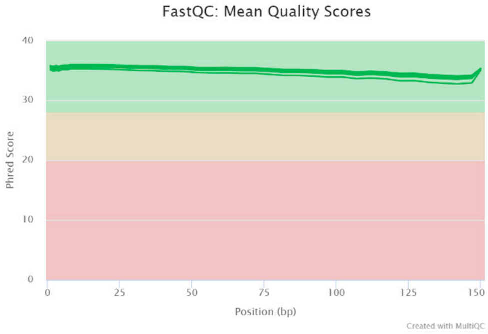

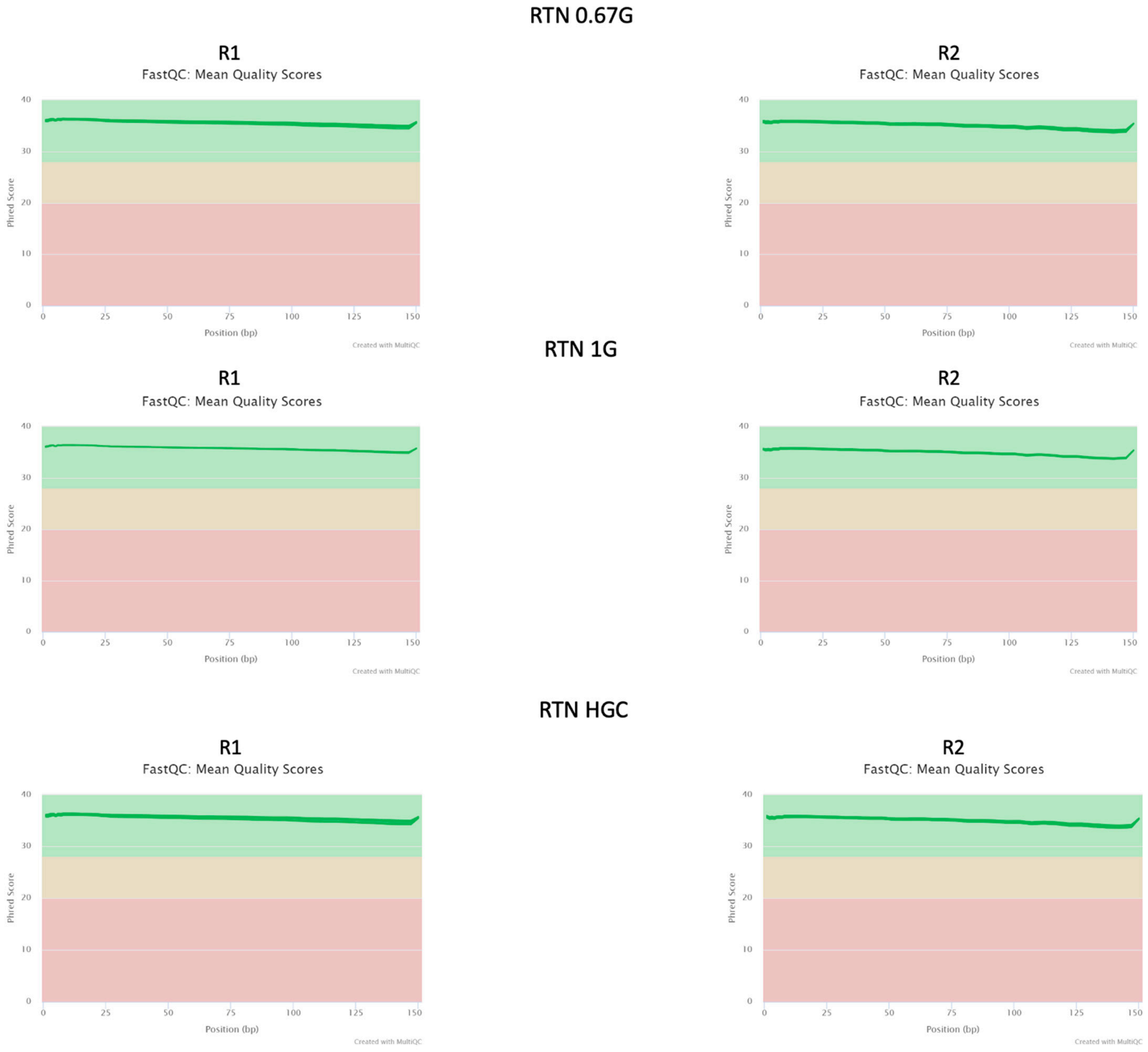

(b) MultiQC Analysis RTN

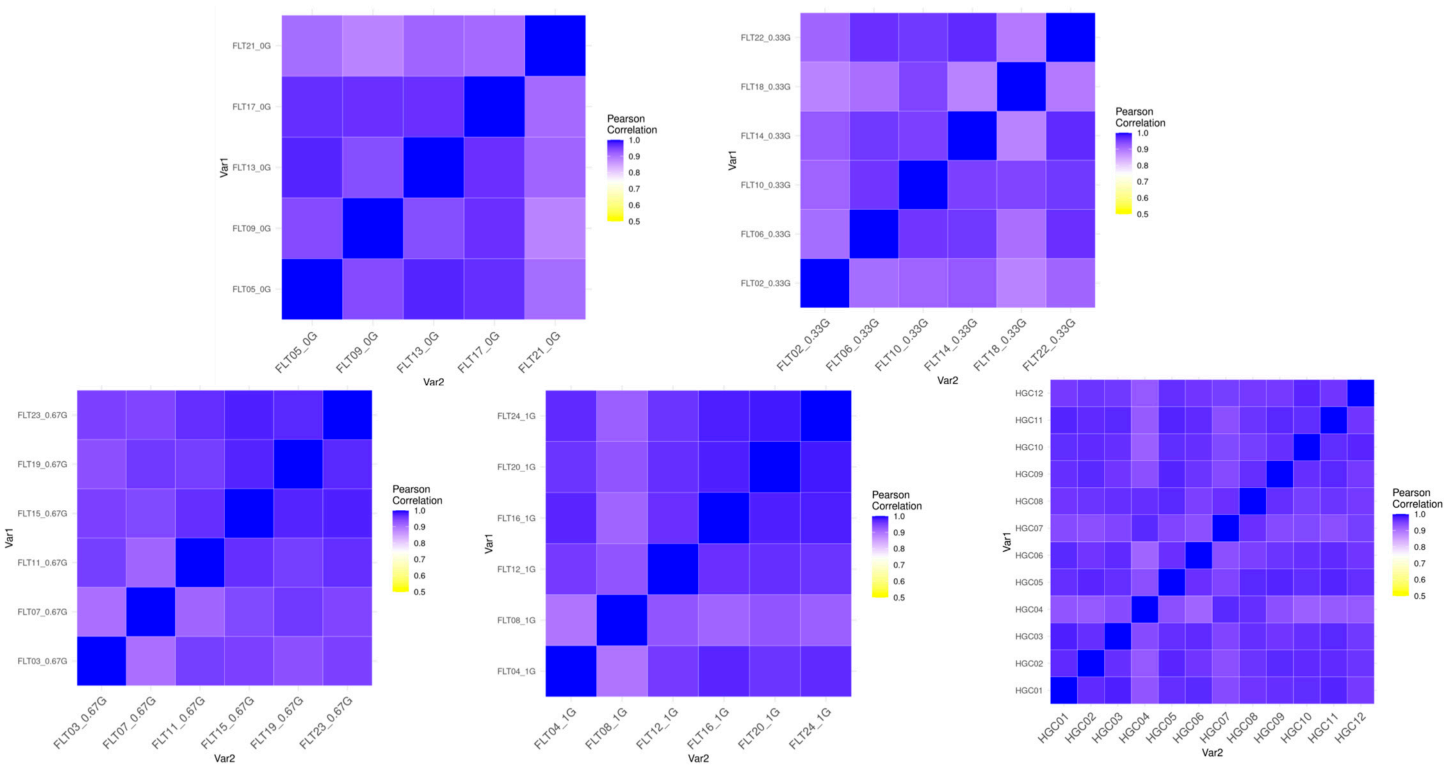

(c) Replicate correlations ON

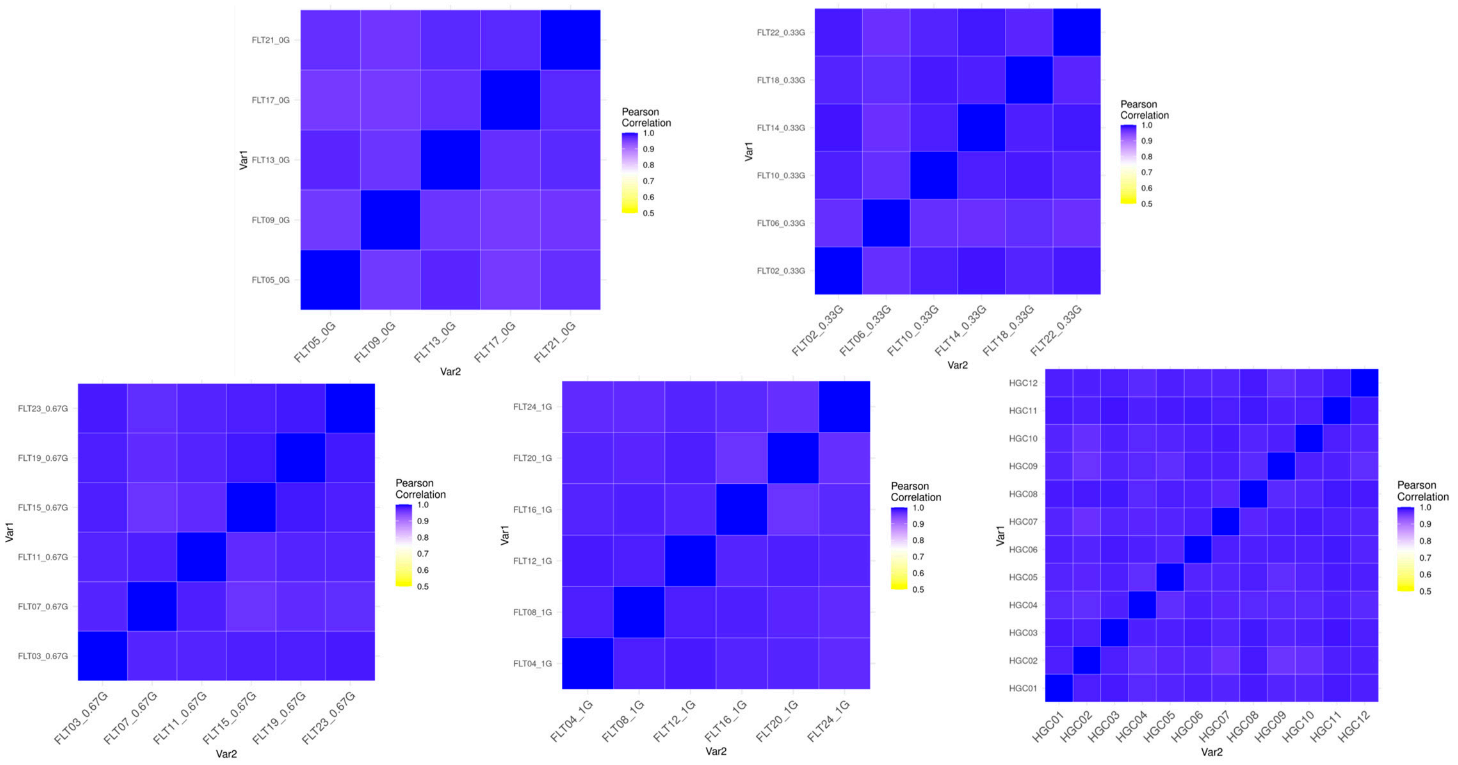

(d) Replicate correlations RTN

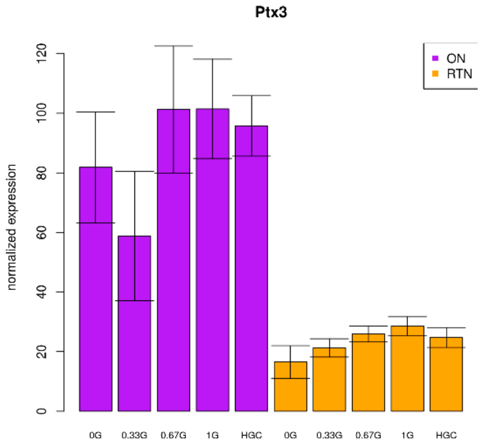

(e)

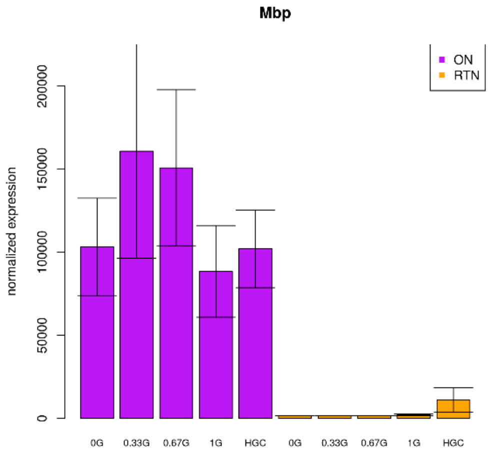

(f)

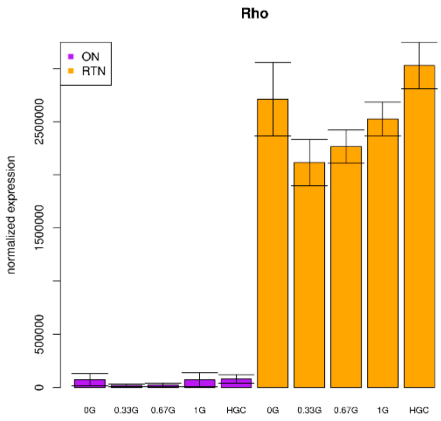

(g)

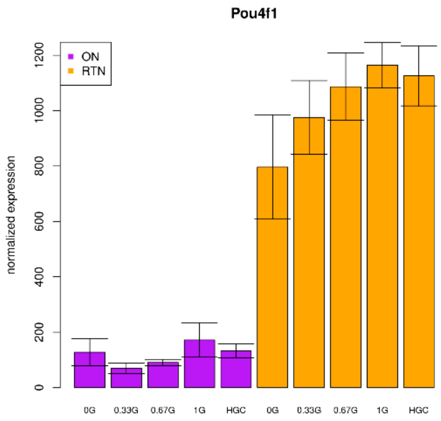

(h)

Figure S1. RNA-seq quality control. (a),(b) Per-base mean quality scores of all replicates of the indicated treatment group and sample type from MultiQC using FastQC. For each group, separate plots of mate1 (R1) and mate 2 (R2) reads are shown. **(c),(d)** Heatmaps visualizing Pearson's correlation coefficient between the natural logarithm of 1 + normalized expression of replicates of the indicated treatment group and sample type. **(e)–(h)** Barplots indicating the mean ± S.E.M. of the normalized gene expression of the indicated gene across replicates of the indicated treatment group and sample type.

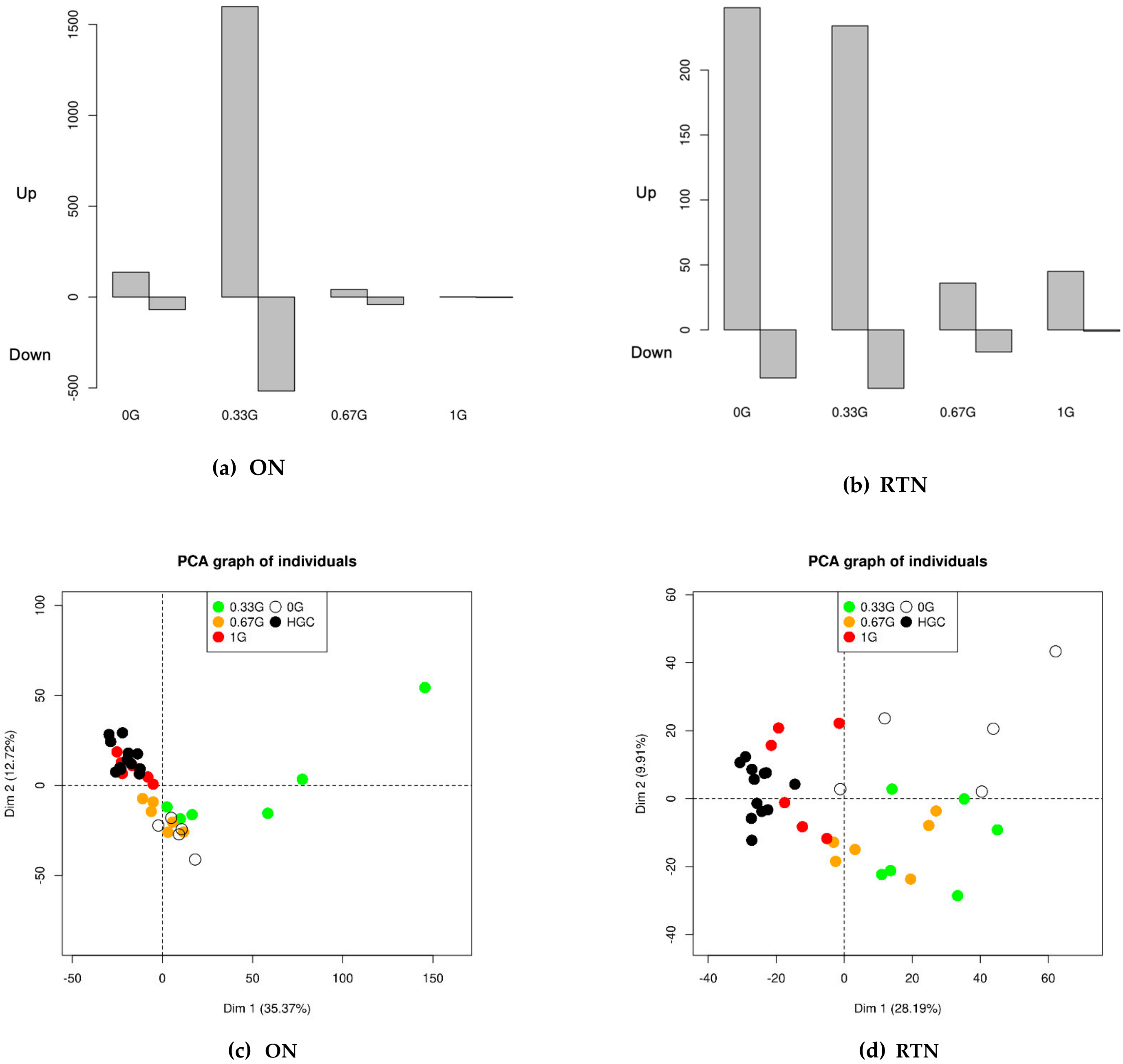

Figure S2. Related to Figure 3. (a),(b) Barplots showing the number of differentially expressed genes in the indicated treatment groups compared to HGC. **(c),(d)** Principal Components Analysis (PCA) plots showing individual replicates of the indicated treatment group and sample type.

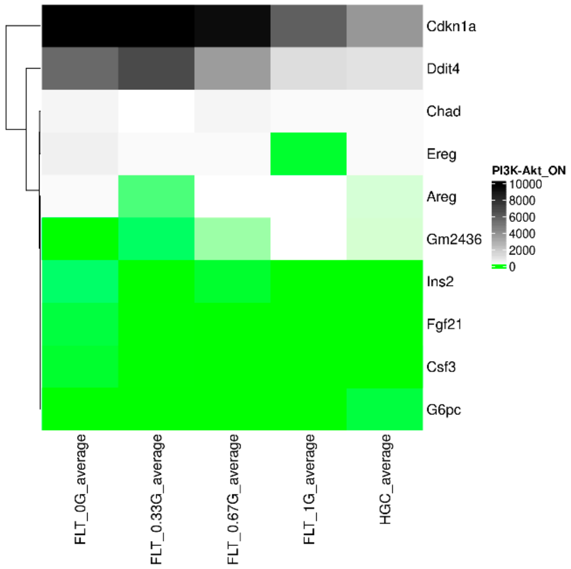

Figure S3. Related to Figure 4. Heatmap of normalized gene expression, averaged over replicates of the indicated treatment groups, of the genes with the top ten magnitude of log2 FC in ON 0G vs. HGC from the PI3k-Akt signaling pathway. Gene symbols are shown to the right of each heatmap. Genes are sorted by hierarchical clustering.
